# Generative AI for Diagnostic Medical Imaging: A Review

**DOI:** 10.2174/0115734056369157250212095252

**Published:** 2025-04-23

**Authors:** Arwa H. Alshanbari, Salha M. Alzahrani

**Affiliations:** 1 Department of Computer Science, College of Computers and Information Technology, Taif University, Taif 21944, Saudi Arabia

**Keywords:** Medical imaging, Generative adversarial networks, Autoencoders, Diffusion models, Transformers, Image-to-text, Text-to-image, Image-to-image

## Abstract

This review provides a comprehensive analysis of recent advancements in generative deep learning (DL) models applied to diagnostic medical imaging, emphasizing their transformative potential in enhancing diagnostic accuracy, reducing radiation exposure, and improving data handling. We explore the architectures, applications, and unique contributions of generative adversarial networks (GANs), autoencoders (AEs), diffusion models, and transformer-based models. The key areas include synthetic data generation for training, text-to-image and image-to-text translation for interpretability, and image-to-image enhancement across imaging modalities. We designed different pipeline architectures presenting basic and advanced generative models specifically designed for medical imaging applications. These include enhanced GAN configurations, such as the multi-layer ML-C-GAN and Temporal-GAN for time-sequenced medical images, and specialized AE-GAN hybrids such as Atten-AE and M3AE, which combine attention modules and language encoding for text-to-image and image-to-text translation. Each pipeline uniquely addresses challenges in synthetic image quality, temporal progression, and accurate caption generation, showcasing their capacity to produce clinically relevant, high-fidelity images across modalities. The discussion highlights these architectural innovations, emphasizing their role in enhancing image synthesis, diagnostic reporting, and patient-specific image interpretation within medical imaging. The review concludes by identifying future directions to refine generative models for clinical applications, ultimately aiming to facilitate more accurate, accessible, and personalized patient care.

## 1 INTRODUCTION

Medical imaging is a cornerstone within healthcare, acting as an indispensable diagnostic instrument for a wide spectrum of diseases and pinpointing various internal deviations such as cancer, aneurysms, and infections. The ability to detect these conditions promptly is crucial, setting the stage for effective therapeutic interventions and enriching our understanding of these complex health challenges [1, 2]. The evolution of medical imaging technologies, notably computed tomography (CT), magnetic resonance imaging (MRI), and X-ray imaging, as well as the retinal fundus, gastroenterology endoscopy, and microscopy, has dramatically improved diagnostic capabilities, facilitating the early detection of illnesses [1, 3, 4]. These advances bolster the potential for successful treatments by revealing detailed insights into the body's structure, such as bone fractures, tumors, infections, and many other medical conditions [3]. Fig. (**1**) shows examples of typical imaging technologies, including CT, MRI, X-rays, retinal fundus, endoscopy, and microscopy, respectively. Despite their great impact on diagnosis, some imaging modalities, such as CT, pose radiation risks, especially for pediatric patients, increasing the risk of brain cancer and leukemia [4]. In practical applications, precise imaging can be more challenging due to the high acquisition cost and longer scanning time [2, 4].

The breakthrough introduction of deep learning (DL) into medical imaging signifies a monumental leap forward [4]. Recent research has illuminated the capacity of these networks to synthesize images that bear an astonishing resemblance to genuine medical photographs, heralding an innovative phase of generative modeling [3, 5]. Currently, DL is being leveraged across a broad spectrum of applications, from classification and segmentation to enhancing image quality and facilitating data augmentation [2, 3, 6].

Additionally, generative models are transforming the diagnostic process in healthcare as they improve both accuracy and efficiency in medical diagnoses [3, 7]. Generative models have the ability to generate synthetic samples that mimic real-world data [8, 9]. This expands the available training datasets and increases the scope of accessible medical data [1, 10]. Text-to-image generative models, in particular, are proving to be incredibly effective in creating and deciphering medical images derived from textual descriptions. This technique involves the precise manipulation of image data, through either the extraction or modification of specific elements based on text, which offers model-centric contextual guidance. Incorporating this method into a transformer layer allows a seamless association to be made between text descriptions and specific image regions, significantly advancing interpretability in text-to-image generation projects on a large scale [6, 10]. Text-to-image models in the medical domain provide detailed insights that enhance patient outcomes. Generative models, both unconditional and conditional, can be further designed for 3D volumetric data, as in a previous study [11], with a primary focus on the brain and heart. They also help patients to better understand their conditions, encouraging more active involvement in their treatment [3, 10].

In the field of medical science, the lack of sufficient data poses a significant challenge to the advancement of DL algorithms, which are critical for accurate diagnosis and patient care [2, 3, 6, 10]. Generative models offer a promising solution to this issue by augmenting and enhancing medical datasets, thereby improving the performance of DL models in tasks such as prediction and diagnosis. Therefore, this study aims to provide a comprehensive review of generative deep-learning techniques within the domain of diagnostic medical imaging. It emphasizes contemporary applications of generative models, their core architectures, and a variety of medical datasets utilized in the field.

The rest of this review is organized as follows. Section 2 outlines the search criteria and research questions that guide the review, providing a structured framework for the subsequent analysis. Section 3 presents an overview of various generative AI models, setting a foundation for understanding their specific applications in medical imaging. Sections 4 through 7 delve into key types of generative models—namely, generative adversarial networks (GANs), autoencoders (AEs), diffusion models, and transformer models—detailing their architectures and roles within diagnostic imaging. In Section 8, the applications of generative deep learning are explored through various techniques, including text-to-image, image-to-image, and image-to-text transformations, which illustrate the practical utility of these models in medical contexts. Section 9 reviews the commonly used datasets crucial for training and validating generative models in this domain. Finally, Section 10 concludes with a discussion on the prospects and challenges associated with implementing generative AI in diagnostic medical imaging.

## 2 SEARCH CRITERIA AND QUESTIONS

In this study, we conducted a comprehensive literature review by utilizing several sources, including Web of Science, ScienceDirect, Springer journals, and ACM journals. Our review primarily covered the updates in the years 2023 and 2024, with some studies from 2022 and one study from 2019 included as well. This review focused on AI journals, conferences, and scientific reports, using keywords such as Text-to-Image in Medical Imaging, Medical Image Synthesis from Text, Diagnostic Image Generation from Text Descriptions, Generative Models for Medical Images and Text, Text-Guided Image Synthesis in Medicine, and Clinical Report to Image Synthesis. Fig. (**2**) illustrates the different generative AI models that contribute to this review, with GANs dominating the research at 36.4%. Fig. (**3**) shows the primary tasks studied in the literature based on generative AI models. It shows that, in this review, most of the recent advancements in the latest studies concentrated on image-to-image generation, followed by text-to-image, and image-to-text.

## 3 OVERVIEW OF GENERATIVE AI MODELS

In recent years, many researchers have demonstrated the ability of DL models to extract essential characteristics from various data types, such as images, for purposes like detecting tumors [3, 12] and diagnosing breast cancer [13] and several diseases of the blood [14]. The progression of DL has led to the introduction of diverse generative models capable of creating high-fidelity images that accurately reflect the distribution of their target datasets [15]. These models are now widely employed for tasks including semantic segmentation, quality improvement, and data augmentation. Various architectures and methodologies for training such models have been developed to support these advancements [12, 16]. Generative AI models, in this regard, include generative adversarial networks (GANs) [8], autoencoders (AEs) [17], diffusion models [6], and others, each contributing to the generation of images across specific domains. This section briefly reviews generative AI models, leaving more details for the later sections.

GANs aim to generate realistic images through competitive training and are commonly employed in medical image synthesis and data augmentation tasks [18]. The adversarial loss introduced by the discriminator offers an ingenious method for integrating unlabeled samples into the training process while enforcing higher-order consistency. This approach has demonstrated its effectiveness in various applications, including domain adaptation, data augmentation, and image-to-image translation [19]. Similarly, AEs compress input data and reconstruct them with minimal loss, focusing on reducing the difference between the input and the output. They are widely used for data denoising and dimensionality reduction [17]. Meanwhile, diffusion models work by gradually adding noise to a simple data distribution and then reversing this noise addition. This approach has allowed for the efficient generation of realistic data [6].

The evolution of generative models has been rapid, leading to the creation of sophisticated systems such as Cycle-GAN [20], Dynamic Memory GAN (DM-GAN [16]), M3AE [21], Latent Diffusion Model (LDM) [10], and Medfusion [22]. These models boast diverse architectural designs and training techniques, impressively enabling the production of highly realistic images across numerous fields, such as medical imaging. This diversity marks a significant milestone in the capabilities of generative models [3, 16]. Cycle-GAN, for example, utilizes GAN architecture to perform image-to-image translation tasks [20], whereas DM-GAN [23] improves upon traditional GANs by addressing multi-layering challenges in text-to-image generation. Cycle consistency is used as a training technique where the model ensures that an input, after being transformed through multiple stages (*e.g*., domain translations), can be reconstructed to its original form. It is commonly used in models such as Cycle-GAN, where data are mapped from one domain to another and then back again. The process enforces consistency between the original and reconstructed data, improving the model's ability to learn meaningful transformations without requiring paired training samples [20].

On the other hand, M3AE leverages autoencoder structures to facilitate functions such as medical visual question answering and image classification [21]. At the same time, LDM [10] and Medfusion [22] showcase how diffusion models have improved image fidelity and multimodal image generation. In addition, transformers in image analysis revolutionize the understanding of complex visual data, enabling superior performance in tasks such as object detection, segmentation, and recognition by leveraging self-attention mechanisms to capture long-range dependencies and intricate patterns within images. Therefore, transformers have been integrated with generative models for image analysis, particularly in medical imaging [2, 4]. Table **1** compares these models, focusing on their architectural designs and training techniques and their usage. The following sections offer comprehensive details of the state-of-the-art generative models along with their main architectures, followed by an exploration of their significant contributions within the medical domain.

## 4 GENERATIVE ADVERSARIAL NETWORKS (GANS) FOR MEDICAL IMAGING

Generative adversarial networks (GANs) are a type of deep generative model that can generate synthetic data [7-9, 11, 19]. The GAN architecture consists of two components, namely, a generator (G) and a discriminator (D), which are trained individually. The primary task of the generator, G, is to learn how to create synthetic data. The discriminator, D, distinguishes between real and fake images produced by the G [3, 18]. Fig. (**4**) illustrates the GAN architecture exemplified to receive medical images as inputs. The generator, G, employs a random noise vector (z) to create a synthetic image, which is then evaluated by the discriminator, D, to ascertain whether it is genuine based on a minimum and maximum loss function [5, 18]. The G aims to generate realistic images to deceive the D by minimizing the loss function. In contrast, the D aims to accurately classify the image as genuine or synthetic by maximizing the loss function. Over time, G and D work against each other, which limits the quality of the images [5, 24]. The Minimax loss function is trained to optimize GANs, as in various studies [3, 18], given as (eq. **1**)

**Table:** 

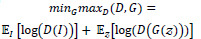	(1)


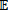
_*1*_[log(*D*(*I*))] represents the expected value over real data distribution *I*, where *D*(*I*) is the discriminator’s output when evaluating a real image. The second part, 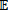
*_z_*[log(*D*(*G*(*z*)))], refers to the expected value over the random noise distribution *z*, where *G*(*z*) represents the synthetic image generated by the generator from the noise vector.

GAN-based models have emerged in research and applications across various domains, leveraging advanced architectures for enhanced image synthesis. Among these, Progressively Growing GAN (PG-GAN), introduced in the study [3, 18], lays the foundation for generating high-resolution images through a methodical, incremental enhancement of the generator and networks. Building on this, Style-GAN, developed in [3, 25] and evaluated in [26], extends PG-GAN’s capabilities. Style-GAN employed adaptive instance normalization (AdaIN), noise injection, and an eight-layer MLP for input transformation. These features enhance image quality, stabilize training, and enable style transfer [3, 5]. Many GAN models specialize in converting images into their synthetic versions [5, 27].

Additionally, different architectures of GANs can transform images into texts [12] and texts into images [24] within specific domains. This highlights their multifaceted utility in generating diverse forms of content. Devasana *et al*. [24] utilized Attn-GAN with Multiple-GAN to automate the creation of high-quality images from textual descriptions. Initially, Attn-GAN produces low-resolution images based on a multimodal conditioned vector. These images are then progressively refined through several steps to yield the final high-quality image, effectively avoiding any degradation in resolution or detail. Thus, multi-GAN models often suffer from a decrease in quality in the last layer. To address this, Azuaje *et al*. [12] developed a tool to translate the textual description to an image using Dynamic Memory (DM)-GAN. DM-GAN enhances the quality of images through a dynamic-memory-based refinement process, which uses textual descriptors to adjust the generation process. By selectively focusing on important words *via* a memory-writing gate and integrating this information into the refinement stages, DM-GAN demonstrates its ability to maintain quality in the first layers, resulting in high-resolution images [12, 23].

The benefit of using GANs to augment data for training has increased the interest in this approach within the medical field [5, 13, 14, 20, 25, 28, 29]. GANs have been utilized for early diagnosis and to save time and expenses. Skandarani *et al*. evaluated the efficacy of different GAN models, including D-CGAN, LS-GAN, W-GAN, Hinge-GAN, SPADE-GAN, and Style-GAN, for medical imaging across MRI, CT, and retina images [5]. The simpler models, DC-GAN, LS-GAN, W-GAN, and Hinge-GAN, share a common architecture that relies on deep convolutional layers with stride layers for the generator and convolutional layers for the discriminator, though they differ in their loss functions. DC-GAN uses an entropy-based loss function, LS-GAN applies mean square error (MSE), WGAN incorporates Wasserstein distance with gradient penalty to improve training stability and minimize mode collapse, and Hinge-GAN optimizes with hinge loss to achieve Nash equilibrium. In contrast, SPADE-GAN used a conditional GAN with a semantic mask input and Wasserstein distance for image-to-image translation. The evaluation of these models utilized FID scores and segmentation accuracy, with the GAN-generated images serving as training data for a U-Net segmentation model. The findings revealed significant differences in performance among the models. Although certain GANs exhibited strong visual fidelity, none were able to fully capture the complexity inherent in medical datasets.

Different imaging modalities, such as microscopes, are used to diagnose diseases such as leukemia and anemia from blood smears. Khan *et al*. [14] introduced a framework leveraging a GAN for the segmentation and classification of blood cells, specifically white blood cells (WBCs), red blood cells (RBCs), and platelets (PLTs). RWP-GAN is based on U-Net architecture that uses spectral normalization and gradient penalty. The generator receives a larger receptive field instead of treating each pixel individually to draw microscopic images to confidence maps, which indicate the pixel probabilities relative to the ground truth. At the same time, the discriminator identifies discrepancies between microscopic images and confidence maps to improve the accuracy of blood cell segmentation and classification and then learns a structured context-aware loss.

Fig. (**5**) compares the D-CGAN, LS-GAN, W-GAN, Hinge-GAN, SPADE-GAN, and RWP-GAN, a simple GAN consisting of a generator and a discriminator. The genuine inputs of G in RWP-GAN are images of blood cells from a microscope, while other models take MRI, CT, and retina images. This figure showcases a variety of basic GAN architectures applied to different types of medical imaging data, illustrating how these models generate synthetic images that mimic real medical images from different diagnostic modalities. On the left, several types of GAN architectures are listed, including DC-GAN, LS-GAN, W-GAN, Hinge-GAN, SPADE-GAN, and RWP-GAN. Each of these GAN models is designed with unique properties and variations in their loss functions or network structures, making them suitable for handling different types of medical images. The input medical images cover a range of diagnostic scans such as MRI cardiac scans of the heart structure, cross-sectional images of the liver obtained through computed tomography (CT), retinal fundus photographs used to diagnose retinal and eye-related diseases, and microscopic images of WBCs, useful for identifying blood disorders. The center section shows the basic GAN architecture, where a noisy vector is inputted into a generator (G), which produces synthetic images. These generated (synthetic) images are then compared to genuine data (real medical images) by a discriminator (D). The discriminator’s task is to differentiate between real and synthetic images, with binary outputs indicating whether an image is classified as real (1) or synthetic (0). This adversarial training allows the generator to improve iteratively, creating increasingly realistic synthetic images that closely resemble genuine data. The bottom section provides examples of synthetic data generated by the GAN models, matching the input modalities. For each modality, cardiac MRI and liver CT images are produced in sequence, demonstrating the ability to generate detailed anatomical structures. The retinal fundus and microscopic WBC images show realistic visuals that could aid in training models for eye and blood diagnostics, respectively.

Meanwhile, Lv *et al*. [20] utilized the cycle-GAN model to generate high-quality synthetic kilovoltage computed tomography (SKVCT) images from megavoltage computed tomography (MVCT) data, thereby improving treatment accuracy and facilitating adaptive radiotherapy. The Cycle-GAN architecture contains two generators in a U-Net structure with convolution or deconvolution layers. The network comprises an autoencoder with an attention gate and nine residual blocks. Cycle-GAN uses two discriminators with convolution layers. Fig. (**6**) illustrates the Cycle-GAN architecture, which is composed of two generators and discriminators, namely, GA, GB, DA, and DB, respectively. GA transforms MVCT images into synthetic kVCT images, whereas GB transforms kVCT images into synthetic MVCT. This is followed by a reconstruction phase: after generator B, the synthetic kVCT images are reverted to actual MVCT images, and after generator A, the synthetic MVCT images are reverted to actual kVCT images. Discriminators DA and DB attempt to differentiate between skVCT and kVCT, as well as sMVCT and MVCT, respectively. This ensures that an image can be converted from an actual image to a fake image and back to a real one, preserving the key characteristics through a cycle of consistency loss.

Gulakala *et al*. [28] and Golfe *et al*. [29] used generative progressive networks that gradually increase the generator and discriminator layers during training. To elaborate, study [28] applied PG-GAN to synthesize high-resolution chest X-ray images for COVID-19 diagnosis. The PG-GAN model takes advantage of synthetic data likely to vary based on the presence or severity of disease indicators in the X-ray images and classifies them *via* CNN. This approach significantly improves the performance of the CNN, enabling a rapid and accurate diagnosis with fewer computational resources than traditional methods. The researchers in a study applied a conditional GAN, ProGleason-GAN, designed for synthesizing prostate cancer histopathological patches with specific Gleason grades. They embedded Gleason-grade information during training to overcome unbalanced datasets commonly seen in prostate cancer classification [29]. The model incorporates stain normalization to enhance visual realism, though it faces challenges in synthesizing certain Gleason grades.

Endoscopy is a representative screening method for diagnosing gastrointestinal cancers. To reduce fatigue among medical staff, Hyun-Cheol *et al*. [25] used an automated approach to the classification of gastrointestinal diseases. They first augmented the data with the style-GAN and star-GAN models to produce a more stable and higher-quality image. Style-GAN, specifically used for its ability to manipulate image styles through a progressive layer and AdaIN, enhances the realism and detail of the generated medical images, while StarGAN trains the GAN to map in different domains within a single generator. A target domain is randomly generated during training, allowing the input images to be transformed into that target. StyleGAN, as shown in Fig. (**7**), introduces a style-based generator with adaptive instance normalization (AdaIN) and noise injection at each layer, replacing the original generator’s progressive layers. Expanding the use of GANs in medical applications, Jiao *et al*. [13] developed the distributed multi-latent code model (which we have named ML-C-GAN) for the rapid and precise generation of breast X-ray images. Their model features a G1 inversion and a G2 generator using a distributed multi-latent code inverse mechanism designed to streamline the data fitting process within the GAN generation. Additionally, the integration of multiple discriminators refined the accuracy of image discrimination, significantly speeding up the generation process and maintaining high image fidelity. The approach showed the ability of their model to supply dependable datasets for diagnostic training purposes.

Schön *et al*. [27] developed the Temporal-GAN, enhancing the capability of GANs for applications in longitudinal medical imaging synthesis. This model involves two generators—G_image_, a traditional generator, and G_temp_, a temporal generator—that together produce continuous and realistic sequences of images depicting temporal developments such as disease progression or treatment effects. Additionally, Temporal-GAN includes two discriminators: D_image_, which differentiates between real and synthesized data; and D_temp_, which ensures volume consistency. This approach uses embedding linear directions in the GAN’s latent spaces for temporal changes, enabling high-fidelity generation with minimal supervision.

Fig. (**8**) compares the ML-C-GAN and Temporal-GAN, an advanced GAN consisting of two generators and two discriminators. This pipeline architecture illustrates the use of advanced GAN models in medical imaging, specifically for generating synthetic medical images from real data inputs.

The left side of the diagram shows the input data for the GAN architecture, including images such as CT coronal lung scans and mammography breast images. These images are provided to various GAN models, such as Temporal-GAN and ML-C-GAN, which are optimized for handling medical imaging data with unique features, such as temporal progression in lung scans. The core architecture in the middle section contains two generators, G1 and G2, which collaboratively synthesize new images that mimic the properties of the input medical data. The process begins with G(z), representing the latent space from which synthetic data are generated, and then passes through both G1 and G2 to produce high-quality synthetic medical images that resemble real-world samples. The GAN architecture creates synthetic data, which are then evaluated alongside genuine data by discriminators D1 and D2. These discriminators aim to distinguish between real (genuine) and synthetic data, thereby refining the generators' ability to produce realistic images. The binary outputs (0 and 1) indicate whether each discriminator classifies the data as real or synthetic. The output section at the bottom shows examples of generated synthetic data. For instance, CT coronal lung images are shown with temporal progression, demonstrating the model's capacity to generate sequences that can reflect temporal changes. This feature is particularly valuable in medical diagnostics, where understanding temporal patterns can assist in tracking disease progression or treatment response. Similarly, mammography images are generated in sequence, potentially aiding in breast cancer screening and other diagnostic purposes.

Table **2** presents a comparative analysis of multiple GAN architectures, such as DC-GAN, LS-GAN, W-GAN, and Cycle-GAN, showcasing their architectures, primary characteristics, and specific limitations in the context of medical imaging applications. Each model is uniquely structured with elements such as entropy, MSE, or hinge loss functions, designed to address distinct challenges in synthetic image generation. For instance, DC-GAN and LS-GAN use stride layers and convolutional layers to generate basic synthetic images but struggle with mode collapse and capturing fine detail. More advanced models such as SPADE-GAN and Cycle-GAN incorporate additional features such as semantic masking and U-Net structures, enabling higher-quality images with detailed segmentation and flexibility across varied medical applications. However, these models are time-consuming and computationally intensive. The RWP-GAN and Pro-GAN provide robust solutions for high-resolution and intricate medical images but come with high computational demands and lengthy training times. Additionally, specialized GANs such as ML-C-GAN and Temporal-GAN are tailored for specific medical needs, such as high-quality breast imaging and capturing temporal changes in sequences, respectively. Despite their advanced capabilities, they face challenges, including the loss of detailed information and limitations in longitudinal data applications. The table below highlights the trade-offs between performance and computational requirements, underscoring the need for careful model selection based on specific diagnostic goals and resource availability in medical imaging contexts.

## 5 GENERATIVE AUTOENCODER (AE) MODELS FOR MEDICAL IMAGING

Traditional autoencoders (AEs) map the input data to a lower-dimensional representation through encoding and then reconstruct the input through decoding to create new data instances. However, in generative AEs, the focus is on learning the underlying probability distribution of the data, which is essential in broader tasks such as image translation and NLP [3, 17]. Fig. (**9**) shows the central architecture of AEs [30, 31] exemplified for medical input data. The encoder compresses the input into a latent space representation, capturing the essential features. In AE, the latent space is a hidden, compressed representation of data where random vectors (inputs) are transformed by the generator to produce realistic outputs, such as images. It captures the underlying patterns and features of the data, allowing for meaningful variations in the generated outputs. Then, the decoder learns to expand this representation back into the original data space or another domain. According to a study [17], the loss function formula of traditional AEs is given by (eq. **2**)

**Table:** 

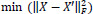	(2)

where *X* represents the input data for the encoder, and *X*' represents the reconstructed output from the decoder. The core AE structure consists of an encoder and a decoder with a latent space in between, forming the main backbone of the model. The encoder extracts meaningful features from the input medical images and maps them into a compact representation in the latent space. The latent space serves as the intermediary, capturing essential data characteristics and enabling the model to generate variations. The decoder reconstructs the features from the latent space, producing synthetic images based on the encoded information.

Variational autoencoders (VAEs) utilize this structure for unsupervised learning, efficiently generating new data that mimic the training set [21, 32]. Yuxiang Wei *et al*. [31] proposed a learning-based encoder for encoding visual concepts into textual embeddings (ELITE) for tailored text-to-image creation, featuring global and local mapping networks for rapid and precise visual concept encoding. VAEs showcase superior fidelity, editability, and speed over traditional methods.

AEs contribute to medical image captioning by learning compressed representations of medical images [21, 30, 32]. Compressed representations generated by AEs have been used to generate descriptive captions, aiding automated diagnosis and facilitating a deeper understanding of visual features relevant to medical conditions and treatments [6, 32]. Beddiar *et al*. [32] combined generative DL and retrieval-based methods to enhance medical image captions (Atten-AE). The generative model generated a new caption using an attention-based encoder–decoder architecture, while the retrieval-based model refined the caption by retrieving the most similar existing caption from the training dataset. The researchers employed a ResNet50 CNN for medical image encoding and an LSTM-based decoder for text generation, enhancing caption quality through attention mechanisms and generative- and retrieval-based methods. However, the low quality of the generated captions showed a negative effect on the final output. On the contrary, Chen *et al*. [21] used a novel pre-training method, the multimodal masked autoencoder (M3AE), to learn effective representations from large-scale medical image–text datasets without needing fine-grained annotations for medical vision-and-language tasks. The M3AE used a transformer-based model for vision (CLIP-ViT) and language (BERT) encoders, processing medical images and texts. A multimodal fusion module integrated visual and text representations with masking ratios based on information density. The models tested on three specific tasks; namely, medical visual question answering (Med-VQA), medical image–text classification, and medical image–caption retrieval for both image-to-text and text-to-image. Wang *et al*. [30] applied an autoencoder-based conditional optimal transport generative adversarial network (AE-COT-GAN) to generate medical images. AE-COT-GAN used AE to obtain a low-dimensional representation of real images and applied semi-discrete optimal transport (OT) to map Gaussian noise to the latent space distribution. A GAN component then enhanced the realism of the generated images. AE-COT-GAN addresses challenges such as mode collapse, producing high-quality images tested on datasets such as DermaMNIST and BloodMNIST.

Fig. (**10**) shows a comparison of Atten-AE, M3AE, and AE-COT-GAN. This figure illustrates the architecture of AE models integrated with GANs for medical imaging applications, showcasing how genuine medical images and captions are processed and transformed to produce synthetic data with captions. On the left side, Atten-AE, M3AE, and AE-COT-GAN model types are used to input genuine images and captions. These models handle diverse image types, including chest radiographs and other medical imagery, to process initial medical data accurately. Both Atten-AE and M3AE used the same dataset, which includes images accompanied by text descriptions. In the Atten-AE model, the text description is entered into the attention model and then into the latent space. After that, the text is generated by the decoder. Recent advancements in unsupervised methods demonstrated success in discovering interpretable directions in GAN latent spaces for natural images. A study extended these techniques to medical imaging by training GANs and VAEs on thoracic CT scans and employing unsupervised approaches to explore latent space directions [33]. The findings revealed non-trivial transformations, such as rotation, and variations in anatomical features, such as breast size, indicating that the models captured 3D structures despite being trained on 2D data. These results suggest that unsupervised methods for latent space exploration in GANs could generalize to VAEs and be effectively applied to medical images [33].

In the M3AE model, the text is fed into a pre-trained BERT model, and then the fusion module takes both the image from the decoder and the text from BERT together to generate the final output. In the AE-COT-GAN model, the image is entered into the encoder and the GAN models. The image is generated by the encoder and entered into the discriminator to be classified as real or fake. The attention module, located above the AE architecture, improves the accuracy and relevance of the generated captions by focusing on specific regions of the image data. This mechanism enhances interpretability by emphasizing clinically relevant features, which is particularly valuable in medical diagnostics. The BERT language encoder receives the genuine captions associated with each input image, encoding textual data to facilitate the generation of coherent synthetic captions. The fusion module combines outputs from the AE and the language encoder, aligning visual and textual data to create accurate synthetic representations. The right side displays the generated synthetic images and captions. For example, a synthetic chest radiograph image is produced alongside a synthetic caption, such as “Chest Radiograph shows Bilateral alveolar…” This output demonstrates the model’s ability to create realistic medical images along with meaningful captions, which can be used for training and diagnostic purposes.

Table **3** summarizes several advanced AE models used in medical imaging, namely, Atten-AE, M3AE, and AE-COT-GAN, highlighting their architectures, strengths, and limitations. Firstly, Atten-AE uses a ResNet50-based encoder with an LSTM-based decoder, incorporating attention and retrieval mechanisms. It is designed to improve captioning accuracy and capture essential image features, making it suitable for generating descriptive medical image captions. However, it struggles with out-of-class images and has a low BLEU score, indicating limitations in generating precise language output. Secondly, M3AE integrates encoders based on ViT and BERT, using a self-supervised model that combines visual and textual data on a large scale. This multimodal fusion allows the model to analyze complex medical images while associating them with text. Its main drawback is high computational demand, which may limit its application in real-time or resource-constrained settings. Additionally, it may not generalize well to all medical fields, restricting its versatility. Thirdly, AE-COT-GAN employs an autoencoder with conditional GAN and Optimal Transport (OT) methods to minimize mode collapse and mixing, thereby producing more reliable synthetic images. However, it is computationally intensive, which could be a barrier to widespread application in routine medical imaging tasks.

## 6 GENERATIVE DIFFUSION MODELS FOR MEDICAL IMAGING

Generative diffusion models are a class of deep generative models that have significantly impacted the field of AI and creativity [6]. Recent research indicates that diffusion models have garnered increasing attention in recent years, surpassing variational autoencoders and GANs in popularity for medical image data augmentation [7]. These models operate by gradually transforming data into a Gaussian distribution through a forward process and then learning to reverse this process, thereby generating new data samples from the noise, as shown in Fig. (**11**). This approach has enabled the generation of high-quality, diverse samples across various domains such as imagery, text, and healthcare, showcasing the models’ wide applicability and transformative potential [6, 15, 34, 35].

The architecture of diffusion models generally follows the methodology of adding noise in the forward process and then training a neural network to reverse it. The reverse process involves a sequence of denoising steps that iteratively reconstruct the original data from noise, producing more stable and accurate outputs. Diffusion models are particularly effective because this iterative denoising allows for precise, high-quality output, especially in image generation tasks [6, 15, 36, 37]. Building upon this, Endo introduced a technique that allowed spatial control over image generation [15]. This method used semantic masks to manipulate attention maps within diffusion models, enhancing user control over the image outcomes without retraining. This masked-attention guidance technique innovatively adjusted the cross-attention maps by altering the noise maps input into the diffusion model, overcoming the challenges associated with direct manipulation.

Similarly, advances in hierarchical vision transformer diffusion models have further enhanced image generation tasks by integrating scene-graph-based semantic layouts with diffusion processes. Swinv2-Imagen leverages Swin-transformer-based U-Net architecture to improve text-to-image synthesis by focusing on local-to-global feature extraction, addressing the limitations found in traditional CNN-based architectures [37]. The model enhances the understanding of relationships between objects in complex scenes, ensuring more coherent and realistic outputs.

At the core of diffusion models, the process can be understood mathematically. According to several studies [6, 26, 36, 37], the forward diffusion process of denoising diffusion probabilistic models (DDPMs), where noise is added step by step, is represented as (eq. **3**):

**Table:** 

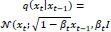	(3)

where *x_t_* represents the noisy data at step *t*, *β_t_* is the variance schedule, and *N* is the normal Gaussian distribution. Over time, noise is added to the data through this process, gradually corrupting the image. To generate data, the reverse process denoises these samples, aiming to reconstruct the original clean data. The denoising step can be expressed as (eq. **4**):

**Table:** 

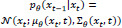	(4)

where *µ*_*0*_(*x_t_*, *t*) is the mean predicted by a neural network, and Σ_*θ*_(*x_t_*, *t*) is the variance. This reverse process iteratively reduces the noise, producing high-quality, accurate outputs​. These models allow for fine control over the generated outputs by conditioning the generation process on additional inputs such as text prompts, masks, or edge information. This makes diffusion models particularly useful for tasks that require precise control and interpretability [6, 10, 36, 37].

In medical imaging, traditional GANs have facilitated the generation of training images. Still, they are often hindered by issues such as mode collapse, vanishing gradients, and convergence, especially with complex medical images [4, 10, 34]. In contrast, recent advancements in diffusion models, such as DDPMs [6, 26], latent DDPMs [22], latent diffusion models (LDMs) [10], conditional DPMs [34], EMIT-DIFF [36], stable diffusion [38], and guided language-to-image diffusion for generation and editing (GLIDE) [35, 39] have demonstrated superior performance over GANs in this domain. Given this, a study evaluated the conditional latent DDPM, Medfusion, specifically designed for medical image generation [22]. Medfusion leverages a pre-trained autoencoder to encode images into a latent space, which is then diffused into Gaussian noise. A U-Net model denoises this latent space, aiming to minimize the noise distribution differences, thereby generating high-quality images through a denoising diffusion implicit model (DDIM).

Furthermore, text-to-image methods often use contemporary diffusion models that include additional conditions that allow for more control over the details of image synthesis and data augmentation. These methods typically use pre-trained language models that encode text inputs into latent vectors [10, 34]. In another study, LDM capabilities were enhanced to synthesize multimodal medical images. Their model integrated CLIP with a diffusion-based, prompt-conditional image generation architecture and an autoencoder, with the inclusion of textual descriptions that directly influence the generation process [10]. The cross-attention mechanism allows the model to focus on specific areas of an image corresponding to textual prompts, enhancing the relevance of generated features. Progressively, denoising images within a perceptual latent space from initialized random noise vectors are decoded back into images that refine the output based on the input structural binary masks. Another study [34] introduced a conditional diffusion probabilistic model (DPM) that combines stable diffusion techniques with image and text-based conditioning. The conditional DPM was designed for synthesizing realistic prostate MR images, employing text- and image-based conditioning to generate one type of MR image based on another.

Meanwhile, Nurbanu Aksoy *et al*. [41] proposed a controllable diffusion model, EMIT-DIFF. EMIT-DIFF integrates traditional diffusion processes with an edge-guided mechanism using text-guided diffusion models. The architecture was designed to enhance medical image segmentation by leveraging the edge information of objects during a text-guided diffusion process within medical images. The process included pre-training on RadImageNet and fine-tuning task-specific datasets to improve generalization and performance.

Some previous studies reported the performance effectiveness of their models based on stable diffusion [22, 34, 36]. Stable diffusion is a high-performance model that rapidly produces higher-quality images that consume fewer resources and require less memory [40]. Xu *et al*. [38] used the stable diffusion model to fine-tune small datasets and generate text-conditioned images specific to medical conditions. Features were extracted from the data, augmented, and fine-tuned again while maintaining medical relevance. Then, an image corresponding to the text was created using denoising diffusion implicit models (DDIMs) and K-samples. Studies conducted by Shavlokhova and Kather [35, 39] highlighted the promising potential of the GLIDE model in medical imaging based on stable diffusion. GLIDE is a text-conditional model fine-tuned with dermoscopic images. It incorporates language processing capabilities to guide image synthesis. Cross-attention layers that integrate text and visual information allow images to be synthesized that closely match text descriptions [35, 39]. Kather *et al*. suggest that GLIDE can effectively capture crucial medical concepts even without specific training, particularly in the fields of oncology and histopathology [39]. Shavlokhova *et al*.’s work built upon this by fine-tuning GLIDE on dermatological images, which boosts the model's performance by incorporating synthetic images [35]. While GLIDE may hold promise for radiology applications if fine-tuned for the domain, it cannot match the quality of specialized generative AI models.

Table **4** provides a summary of various diffusion models applied in medical imaging, detailing their architectures, features, and limitations. Models such as Medfusion and Pathology CDPM utilize conditional architectures to enhance high-fidelity image synthesis across multiple modalities, though they face challenges such as resolution constraints and limited anatomical focus (*e.g*., central prostate synthesis in Pathology CDPM). Controllable DDPM and EMIT-DIFF incorporate text-guided image synthesis with cross-attention mechanisms to achieve multimodal control and realism, although they are hindered by low sampling speeds. TDASD and GLIDE are designed to handle data scarcity effectively; however, they require extensive fine-tuning, especially on limited datasets.

## 7 GENERATIVE TRANSFORMER MODELS FOR MEDICAL IMAGING

The impact of transformers has extended to medical imaging, achieving state-of-the-art results in different tasks [2]. Recently, transformers significantly influenced medical image analysis for diagnosing diseases through CT and X-ray images, and have excelled in image classification and synthesis, showcasing their versatility and effectiveness across domains [2, 4]. Transformers employ an attention mechanism that prioritizes significant features while disregarding less relevant ones. They utilize an encoder–decoder structure to manage complex sequential tasks adeptly, capturing deep contextual relationships within the data. This enables precise output generation, such as translations [2]. Fig. (**12**) shows an illustration of transformers. According to [2], the mathematical formulation of the attention mechanism within transformers, which underpins their effectiveness, is given by (eq. **5**):

**Table:** 

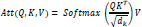	(5)

where *Q*, *K*, and *V* denote the query, key, and value, respectively, and *d_k_* is the dimension of the keys. This mechanism computes the attention scores that dictate the influence of different parts of the input data on each other, enhancing the transformer's ability to focus on pertinent features necessary for accurate medical diagnosis. Transformers have been employed recently for the generation of images from textual medical reports. CLIP, is a multimedia conversion tool that can identify images based on textual descriptions without the need for image tagging [2]. The effectiveness of the CLIP technique also indicated a pathway toward developing more precise and resilient approaches. Several research efforts have employed CLIP as a pre-trained large language model to enhance their studies [10, 15, 21, 40]. Leveraging the capabilities of transformers, Barreto *et al*. [1] developed a model combining transformers with CNNs to create textual descriptions for medical images. The model featured two interconnected transformer blocks: an encoder block that encodes CNN features and a decoder block that processes text tokens and outputs from the transformer encoder. Typically, a CNN first processes the image to create a set of feature maps that encode visual information, which are then fed into a transformer. The transformer uses self-attention mechanisms to analyze and generate text based on the contextual relationships between the encoded features. Akso *et al*. [41] also utilized a transformer-based encoder–decoder network, integrating it with patient demographic data to generate personalized radiology reports from chest X-ray images. The network combined visual features extracted from CXRs *via* a CNN with embedded patient demographic information (such as age and gender) to the transformer. The transformer's encoder–decoder structure processed this combined input, using the attention mechanism to focus on relevant features and generate detailed, context-rich radiology reports. This integration of multimodal data allowed the system to produce more personalized and clinically relevant reports. In another context, Wang *et al*. [42] focused on employing a conditional GAN enhanced with a Spatial-Intensity Transform (SIT) for medical image-to-image translation tasks. The CGAN architecture typically consists of a generator and a discriminator that are trained simultaneously in an adversarial process. The generator uses a transformer-based network to map input images (such as lower-quality MRIs) to transform outputs, while the discriminator evaluates the authenticity of the generated images against real ones. The SIT component is integrated into the generator, providing a structured way to modify both the spatial arrangement and intensity values of the pixels, thus ensuring that the generated images retain physiological accuracy and visual fidelity. This setup allows for precise modifications to be made to image attributes such as age or disease progression while maintaining a realistic appearance.

Table **5** highlights different transformers and GAN-based models used in medical imaging, each designed to tackle specific diagnostic challenges. TDCNN-Transformers [1] combine transformer architectures with autoencoders and CNN mapping features to generate textual descriptions from medical images, aiming to generalize across multiple imaging modalities. However, they face limitations in data availability and the quality of generated descriptions. RRG-Transformer integrates patient demographic data with imaging features to produce radiology reports, but its effectiveness is limited by the availability of accurate demographic data essential for model performance [41]. GAN-SIT uses a conditional GAN with spatial-intensity transformations to visualize age and stroke-related changes in brain scans, aiding in the assessment of stroke severity. Despite its utility, this model requires extensive fine-tuning to ensure accurate and relevant output [42].

## 8 APPLICATIONS OF GENERATIVE DEEP LEARNING IN MEDICAL IMAGING

Generative DL models offer innovative solutions to some of the medical imaging field’s most persistent challenges. As healthcare providers increasingly rely on imaging diagnostics, the need for enhanced image quality, reduced radiation exposure, and efficient data handling becomes more critical [2, 4]. These models facilitate a range of applications, from synthetic data generation, which mitigates the risks associated with direct patient imaging, to advanced techniques such as image reconstruction [4].

The potential of GANs, for instance, to generate high-fidelity medical images enables clinicians to train with extensive image datasets without compromising patient privacy [18]. Meanwhile, autoencoders excel in denoising and dimensionality reduction, crucial for enhancing image quality and speeding up computational processes [17]. Further, diffusion models offer a cutting-edge approach to synthesizing detailed medical images through stochastic processes, providing tools that adaptively improve with more data [6]. Similarly, transformer models contribute by harnessing their robust attention mechanisms to model complex dependencies within imaging data, enhancing tasks such as text generation, thus enabling more accurate diagnostics and treatment planning [2]. This section highlights the diverse applications, such as text-to-image, image-to-text, and image-to-image, of these advanced generative models in overcoming the problem of traditional imaging techniques, improving diagnostic accuracy, and personalizing patient care.

### 8.1 Text-to-Image

Text-to-image translation is one application of generative models with significant benefits in various fields. These models have made marked strides in generating high-quality images from textual descriptions, which is crucial for applications ranging from creative arts to computer-aided design [24, 31]. These techniques allow for the automatic generation of visual content based on text, which can help those who struggle with visualization from text alone, enhancing understanding and accessibility. Additionally, text-to-image models are making significant contributions in fields such as medicine by providing immediate and accurate visual representations of descriptive content, thus aiding in faster decision making [37].

As can be seen in Table **6**, the integration of text-to-image conversion technologies in the medical field has significantly advanced medical imaging by allowing for the creation of accurate and detailed visualizations from textual descriptions [21]. Text-to-image generative AI models have enhanced diagnostic accuracy, training, and planning in various medical applications by producing synthetic images visually and clinically representative of real medical scenarios [38]. By learning from cross-modal domain knowledge, Chen *et al*. introduced multimodal masked autoencoders that enhance comprehension in medical vision-and-language tasks [21]. The model achieves state-of-the-art results on downstream tasks such as medical visual question answering and image–text classification, proving its efficacy in improving medical image and text comprehension.

Recently, diffusion models have gained attention for their ability to generate high-quality images with fine-grained control during inference [15, 34, 36, 38, 39]. The researchers in [15] used diffusion models for spatially controlling text-to-image generation without further training in precise feature placement, thus optimizing medical imaging applications and demonstrating more accurate spatial control than baseline methods. Similarly, a method was developed in to synthesize bi-parametric prostate MRI images by adapting to both pathology and imaging sequences using a stable diffusion model. The authors of [34] developed EMIT-DIFF, a text-guided diffusion model that enhances medical image segmentation, addressing the scarcity of high-quality labeled medical data by generating synthetic images that adhere to medically relevant constraints, significantly improving segmentation accuracy in various medical imaging modalities [34]. The researchers in explored the application of the GLIDE model in medical imaging, pointing out its potential utility after domain-specific fine-tuning, which could revolutionize text-conditioned medical image processing tasks [35, 39]. In another study stable diffusion models were used to overcome data scarcity and enhance the quality of medical training and diagnostic models [35, 38]. The generated images showed improved performance when augmented with synthetic data. The researchers in [39] noted that the model sometimes produced mixed results when generating images without domain-specific training.

### 8.2 Image-to-Text

In the context of the increasing importance of medical images, the development of generative models capable of automatically and efficiently producing descriptive texts for medical images is of paramount importance. This approach simplifies the interpretation process for patients and reduces the workload of healthcare providers. As seen in text-to-image translation, the model M3AE, introduced by Chen *et al*. has the ability to produce texts from images to enhance tasks such as classification and retrieval [21]. Similarly, Beddiar *et al*. AEs in generative and retrieval captions to improve the accuracy and relevance of medical image descriptions, thereby enhancing the practical utility of image captions in medical settings [32]. This approach leverages the strengths of both generative and retrieval techniques and significantly improves the relevance and accuracy of the captions produced.

As demonstrated in Table **7**, Barreto *et al*. [1] and Aksoy *et al*. [41] both leverage the synergy of CNNs and transformers to advance medical image analysis but focus on different enhancements and applications. CNNs are employed for extracting key features from medical images, which are then processed by transformers to generate textual descriptions that accurately reflect complex medical details [1]. Taking this integration a step further [41], non-imaging data, such as patient demographics, were integrated with medical images in a transformer-based model. Its aim was to capture visual features from chest X-ray images using CNNs and enrich the text-generation process with additional patient-specific information. The result was the production of more comprehensive and personalized radiology reports that offered deeper insights.

### 8.3 Image-to-Image

Image-to-image translation is helpful in several fields, enabling the conversion of one image format to another and enhancing applications in areas such as medical imaging, image editing, and even video enhancement. These models allow the conversion of images to higher resolution or improve image quality in terms of brightness, contrast, and noise levels [3, 16]. Furthermore, in the medical field, these techniques help convert different imaging modalities (*e.g*., MRI to CT) or create detailed visual representations from less clear images, facilitating more accurate diagnoses and effective treatment planning [2, 3]. Table **8** shows the different models for image-to-image translation tasks.

GANs have been effectively applied in various medical imaging contexts to enhance diagnostic accuracy and imaging quality [5, 13, 14, 20, 25, 27]. In an experimental study [5], generative networks were evaluated across different modalities, such as cardiac MRI, liver CT, and retinal images, to understand their ability to produce realistic medical images. The researchers found that some advanced generative network architectures could produce high-resolution images that could fool professionals. Additionally, the introduction of the dm-GAN framework employs multi-latent code inversion and multi-discriminator structures for the rapid and accurate generation of breast X-ray images, showing significant enhancements in both speed and image accuracy, with improved PSNR and FID scores [13]. The researchers in a study utilized Cycle-GAN to enhance MVCT images to produce kVCT images [20]. Further applications demonstrated the versatility of GANs in medical imaging. In a study, a framework was developed for the segmentation and classification of blood cells, significantly improving the accuracy over traditional methods [14]. In a study on endoscopic image classification, GANs were used for data augmentation, demonstrating how synthetic images can enhance model performance when data are scarce [25]. Schön *et al*. introduced a novel approach in GANs by embedding temporal changes directly into the model, facilitating the synthesis of longitudinal medical images to track disease progression [27].

In an attempt to leverage competitive GANs and overcome common problems such as mode collapse, Wang *et al*. used AE-COT-GAN, an autoencoder, to capture a low-dimensional multi-dimensional set of real images, applied with a competitive generative network to efficiently generate new realistic medical images and enhance the diversity and quality of synthetic medical images [30]. Generating meaningful medical data is challenging due to the complexity of organ appearance. Since GANs suffer from instability during training, Mueller-Franzis *et al*. compared Medfusion, a conditional latent DDPM, to GANs, showing that Medfusion exhibited superior performance in terms of diversity and fidelity compared to GANs, offering fewer artifacts and more realistic medical image synthesis [22].

Transformers have emerged in an attempt to improve the robustness and reliability of medical image translations, especially in circumstances where traditional models may present some drawbacks. Wang *et al*. introduced Spatial-Intensity Transforms (SITs) to improve the quality of medical image translation by constraining generators to produce smooth spatial transformations combined with sparse intensity changes [42]. This approach enhances the fidelity of translated images and provides a better interpretation of the physiological changes captured in the images, which makes it valuable for applications such as predicting disease progression or visualizing patient-specific changes due to aging or disease severity.

## 9 DATASETS

In the literature, many datasets have been used to train and test generative models. Some have been tested on public datasets, whether images or texts. This section focuses on evaluating models using medical data that serve as foundational resources for training and validating DL models, with significant applications in diagnostic and treatment processes. These datasets typically comprise various image modalities, such as MRI, CT, and PET scans, which aid in the detailed examination and understanding of various diseases and conditions. Utilizing generative models enhances medical image synthesis, aiding in tasks such as generating synthetic images and classification for training without privacy concerns [3, 4]. However, collecting medical data is challenging due to privacy concerns and high-quality annotations. The exchange and use of medical images are complicated by the sensitive nature of health information. Furthermore, the quality of medical datasets often depends on the accuracy of the annotations, which can vary significantly based on the annotator’s expertise and experience. Data scarcity and imbalance affect the model’s ability to learn effectively, potentially leading to biased or underperforming models [3, 4, 43]. The datasets listed in Table **9** have been extensively used across various studies to train and evaluate generative models. These models have been tested on both public and private datasets, covering a range of imaging modalities and medical conditions. The table provides a comprehensive overview of these datasets, detailing their content, the specific medical imaging modalities they employ, the organs they focus on, the structure of their training and testing splits, and the tasks they are typically used for in research.

## CONCLUSION AND FUTURE WORK

In conclusion, the integration of generative deep learning models into diagnostic medical imaging has marked a transformative step forward, enabling unprecedented advancements in image synthesis, quality enhancement, and interpretability. Through architectures such as GANs, AEs, diffusion models, and transformers, generative models have shown considerable promise in applications such as synthetic data generation, denoising, modality translation, and text-guided image creation. These innovations directly contribute to addressing key challenges in medical imaging, including data scarcity, privacy concerns, and the need for high-resolution, interpretable visuals that help both clinical decision-making and patient understanding. The specialized architectures discussed, such as ML-C-GAN and Temporal-GAN, demonstrate the potential to handle complex, domain-specific imaging requirements, such as temporal progression and patient demographics, which are crucial for tracking disease progression and personalizing treatment. However, despite these advancements, challenges remain. Computational complexity, extensive data requirements, and model stability issues, such as mode collapse and fine-tuning demands, limit the deployment of these models in real-time clinical settings. Additionally, the reliance on high-quality, diverse training data across multiple modalities highlights the need for more robust data acquisition and curation methods.

To address the current limitations and enhance the deployment of generative deep learning models in clinical settings, several key areas of future work are identified. First, developing more efficient model architectures and optimization techniques remains crucial. Techniques such as model pruning, quantization, and distillation could significantly reduce the resource demands of these complex models, making them more suitable for real-time clinical applications and easier integration into existing healthcare infrastructures. Furthermore, given the importance of interpretability in medical diagnostics, future research should aim to make model output more understandable and clinically actionable. This includes the development of attention-based mechanisms and explainable AI methods specifically tailored to generative models. Ensuring that the synthesized data align with clinical reasoning and can be adapted to patient-specific contexts will increase their utility in practical settings. Advanced architectures like Temporal-GAN and GAN-SIT have demonstrated their value in handling temporal data, which is crucial for tracking disease progression. Future models should expand on this by incorporating longitudinal patient data, which would enable the models to provide insights into population-level trends while retaining the accuracy needed for individual patient diagnoses. Enhancing these models to adapt to multi-patient datasets could also facilitate a broader application in diverse medical scenarios, further personalizing treatment options and improving patient care. Recent years have witnessed swift and continuous advancements in surgical technology, marked by groundbreaking innovations. Among these, the integration of the Internet of Things (IoT) into surgical practices stands out as a transformative milestone. Consequently, the fusion of IoT with generative AI models in medical imaging emerges as a promising avenue for future exploration and development [44].

## Figures and Tables

**Fig. (1):**
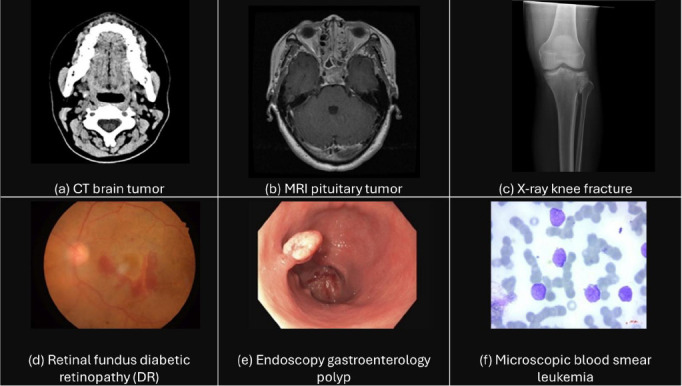
Imaging techniques for different medical conditions: (**a**) CT scan showing the brain tumors, (**b**) MRI scan showing pituitary tumor, (**c**) X-ray scan showing the knee fracture, (**d**) Retinal fundus showing diabetic retinopathy from IDRiD dataset, (**e**) Endoscopy scan showing gastroenterology polyp retinopathy from Kvasir dataset, and (**f**) Microscope scan showing acute lymphoblastic leukemia.

**Fig. (2):**
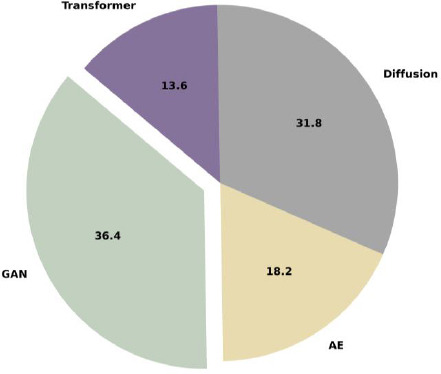
Distribution of literature studies by generative AI models.

**Fig. (3):**
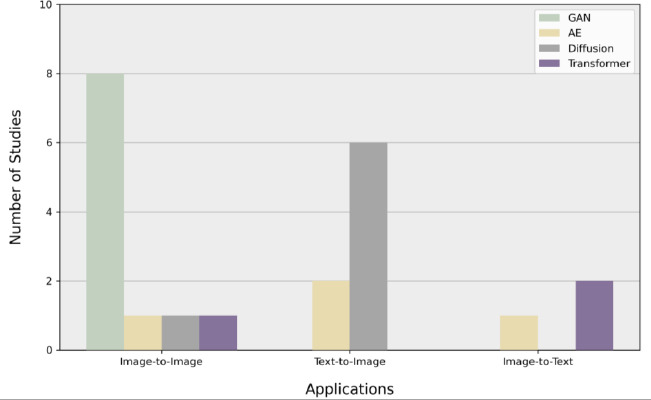
Distribution of literature studies across different generative AI models incorporated in this review.

**Fig. (4):**
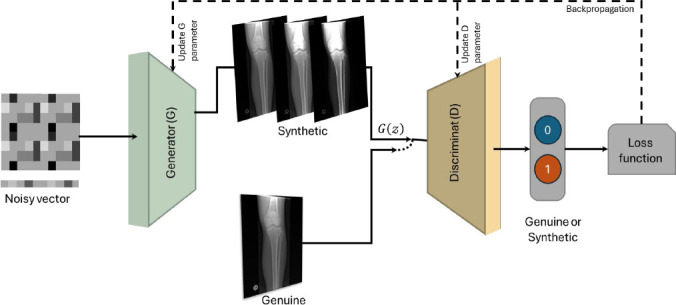
General architecture of generative adversarial networks (GAN) for medical imaging analysis and diagnosis.

**Fig. (5):**
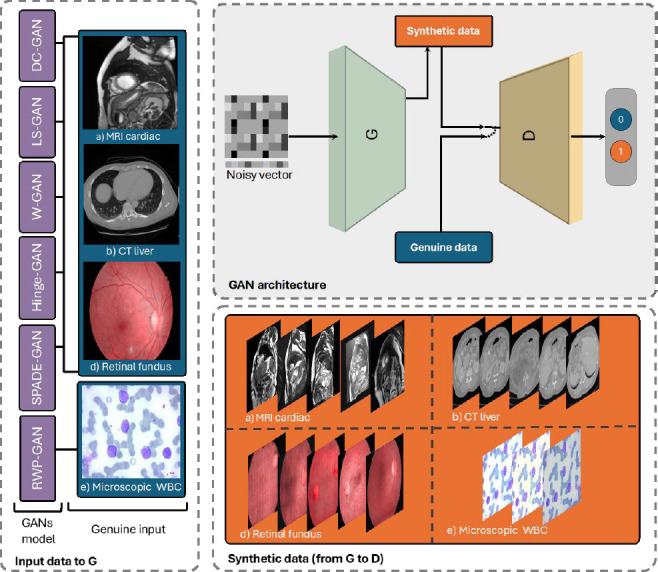
Different architectures of GAN models in medical imaging. The left part represents different medical images used as genuine input data for D training. The upper part represents the fundamental operation of a GAN. The lower represents synthetic data generated by the GAN along.

**Fig. (6):**
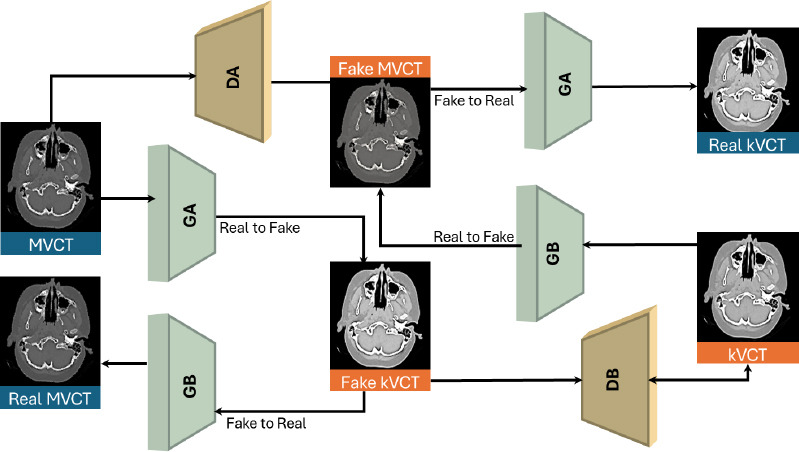
Cycle-GAN-based training architecture for synthesizing high-fidelity kVCT images from MVCT scans in medical diagnostics (redesigned from [20]).

**Fig. (7):**
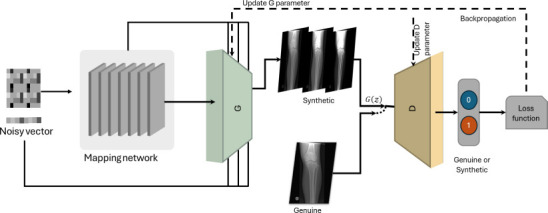
Style-GAN-based training architecture for generating high-quality synthetic medical images from real image data (redesigned from [3, 5]).

**Fig. (8):**
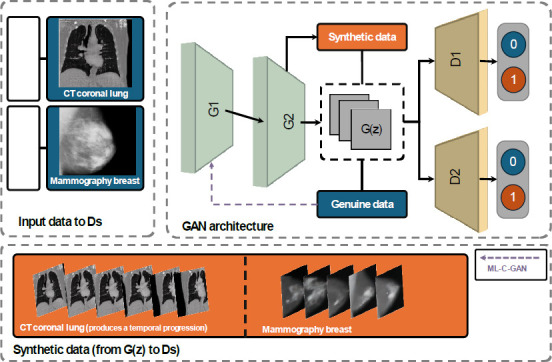
Advanced GAN models in medical imaging. The upper-left part represents different medical images used as genuine input data for D training in temporal-GAN, while ML-C-GAN takes the input for G1 and D. The upper-right part represents the GAN architecture which includes two G and two D. The lower part represents synthetic data generated by the GAN.

**Fig. (9):**
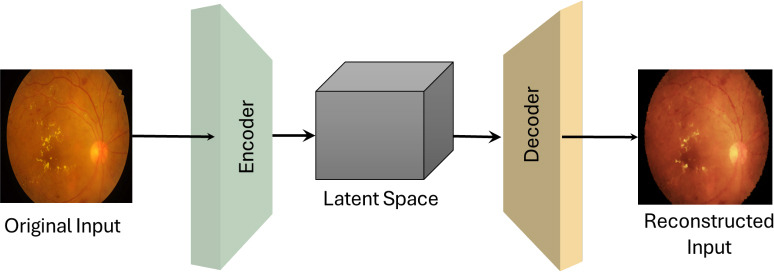
General architecture of auto-encoders networks (AEs) for medical imaging analysis and diagnosis.

**Fig. (10):**
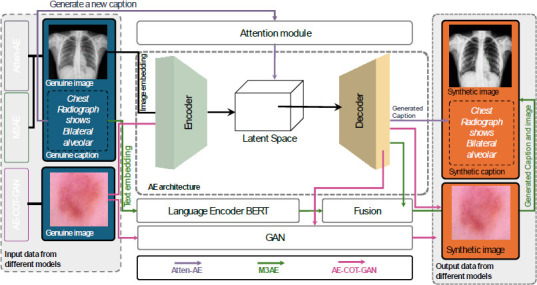
AE models in medical imaging. The left part represents various medical images utilized as genuine input. Inputs of the Atten-AE and M3AE models include a chest radiograph accompanied by a genuine caption. The AE-COT-GAN model provides a genuine image of skin pathology. The middle part represents the core AE architecture which includes encoder and decoder. The right part represents the output data from different models: Atten-AE produces synthetic captions, M3AE produces synthetic outputs where both the image and the corresponding medical caption are generated, and AE-COT-GAN produces synthetic images.

**Fig. (11):**
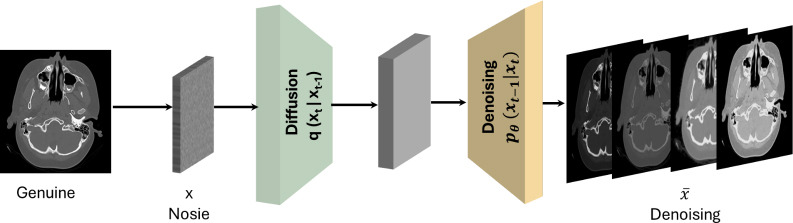
General architecture of generative diffusion networks for medical imaging analysis and diagnosis.

**Fig. (12):**
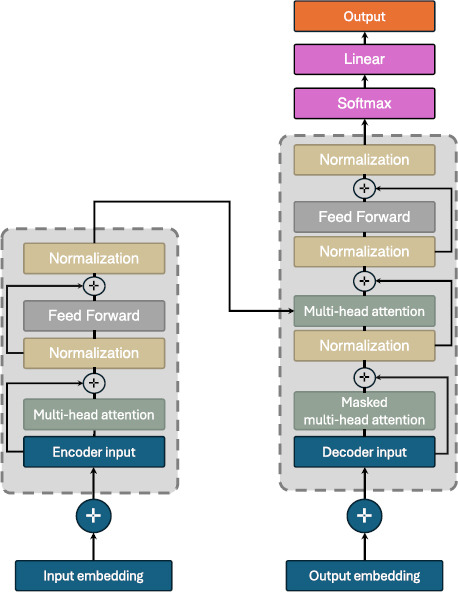
General Architecture of Transformers which can be used for medical image analysis.

**Table 1:** Comparison of generative AI models used in various domains in terms of their architecture, training techniques, and generation/application usage.

**Generative Model\Refs.**	**Architecture**	**Training Techniques**	**Usage/Application**
Cycle-GAN [20]	GAN with two generators and two discriminators. Structure of generator is U-Net with residual blocks and attention gates.	Adversarial training, cycle consistency, identity mapping losses, and gradient magnitude loss.	Image-to-image
DM-GAN [23]	Modified GAN with dynamic memory to refine image layers using textual cues.	Adversarial training with memory mechanisms for iterative refinement.	Text-to-image
M3AE [21]	Multi-modal encoders with transformer for vision and BERT for language, and a multi-modal fusion module.	Self-supervised learning, without details annotation, with different masking ratios.	Medical visual question-answer, classification, Image-to-text, Text-to-image
LDM [10]	Latent space for noise, followed by denoising using U-Net with cross-attention.	Diffusion process with text and structural masks.	Text-to-image
Medfusion [22]	Conditional latent denoising diffusion probabilistic models (DDPM) with U-Net with cross-attention.	Variety of loss functions to enhance the quality and diversity of generated images.	Image-to-image
Transformer [1]	Transformer with Convolutional Neural Networks (CNNs).	Combining NLP and CNN for feature extraction from images.	Image-to-text

**Table 2:** Overview and comparison of various GAN architectures applied in medical imaging, detailing model structure, key characteristics, and limitations.

**Model**\Refs.	**Architecture**	**Characteristics**	**Limitations**
DC-GAN [5]	− Simple GAN with entropy loss function. − Generator uses stride layers. − Discriminator uses convolutional layers.	− Generate synthetic image like MRI and CT. − Used as a baseline model for comparison.	− Mode collapse issue. − Less effective at capturing fine details.
LS-GAN [5]	− Simple GAN with mean-square error (MSE). − Generator uses stride layers. − Discriminator uses convolutional layers.	− Using a MSE loss function. − Solve the vanishing gradient problem.	− Mode collapse issue. − Less effective at capturing fine details.
W-GAN [5]	− Simple GAN with Wasserstein distance, gradient penalty. − Generator uses stride layers. − Discriminator uses convolutional layers.	− Stabilizes training. − Reduces the mode collapse. − Generate a higher-quality image.	− High computational. − Fine-tuning of hyperparameters.
Hinge-GAN [5]	− Simple GAN with hinge loss. − Generator uses stride layers. − Discriminator uses convolutional layers.	− Using a hinge loss function. − Solve the vanishing gradient problem. − Reduces the mode collapse.	− Struggle with complex medical dataset.
SPADE-GAN [5]	− Conditional GAN − Semantic mask input − Wasserstein distance	− Generate high-quality images. − Output aligned with input semantic masks.	− Time consuming process
RWP-GAN [5]	− U-Net-based GAN. − Generator with 8 encoding and 7 decoding blocks. − Discriminator with 3 encoding blocks.	− Pixel-level analysis − Handling high-variability data. − Excellent results in segmenting.	− High computational for small and intricate objects like platelets. − Requires extended training for fine details.
Cycle-GAN [20]	− 2 generators, U-net structure, convolution/deconvolution layers. − Autoencoder, attention gate, 9 residual blocks. − 2 discriminators with convolution layers.	− Ability to work with unpaired datasets. − Highly flexible and useful in medical applications.	− Small training dataset. − Noise reduction. − Soft tissue contrast. − Metal artifacts.
PG-GAN [28]	− Progressively layers for generator and discriminator. − Adversarial training with instance normalization.	− Generates high-resolution images. − Improves stability	− Computationally intensive. − Long training times
Pro-Gleason-GAN [29]	− Progressively layers − Conditional GAN − Incorporate Gleason grade information.	− Synthesize patches with Gleason grades for unbalanced datasets	− High complexity training. − High complexity evaluation.
Style-GAN [5, 25, 26]	− Adaptive instance normalization (AdaIN) − Noise injection in each layer. − 8-layer MLP for input transformation	− Fine-control image attributes. − Generate high-resolution images.	− Time consuming process. − Requires fine-tuning. − Lead to overfitting.
Star-GAN [25]	− Single generator. − Adversarial loss.	− Training across domains.	− High-resolution image issue. − Complex structural changes like polyp removal.
ML-C-GAN [13]	− Two generators: a G1 inversion, and a G2 multi-latent code inverse mapping method. − Discriminator: a multi-discriminator.	− Generates a diversity of high-quality breast images − Faster processing time.	− Loss of breast tissue information. − Time consuming process
Temporal-GAN [27]	− 2 discriminators: traditional and volume consistency. − 2 generators: traditional and temporal.	− Generate sequences of images. − Capture temporal changes.	− Limited to longitudinal data applications. − High computational.

**Table 3:** Overview of advanced autoencoder (AE) models used in medical imaging, detailing model architecture, key characteristics, and limitations.

**Model**\Refs.	**Architecture**	**Characteristics**	**Limitations**
Atten-AE [32]	− Encoding based on ResNet50 − Decoder based on LSTM − Attention and retrieval mechanisms	− Enhanced caption accuracy − Captures important image features.	− Struggled with out-of-class images − BLEU score is low.
M3AE [21]	− Encoders based on ViT and BERT − A multi-modal fusion with co-attention mechanisms − Decoders map image to text	− Self-supervised model − Combines visual and textual in a large scale	− Not generalize well to all medical fields. − Computationally complex.
AE-COT-GAN [30]	− Autoencoder, conditional-GAN − Optimal transport OT	− Minimizing mode collapse − Minimizing mode mixing	− Computationally complex.

**Table 4:** Comparison of generative AI models employing diffusions architecture for medical imaging: architectural designs, characteristics, and limitations

**Model**\Refs.	**Architecture**	**Characteristics**	**Limitations**
Medfusion [22]	− Conditional latent DDPM, U-Net − Autoencoder-based denoising − Loss function: MSE, MS-SSIM and PSNR.	− Used various modalities. − Diverse and high-fidelity images.	− Resolution Constraints − Lack of Component Analysis
Controllable DDPM [10]	− Prompt-conditional architecture. − Pre-trained model (CLIP). − Cross-attention.	− Text-guided multi-modal control.	− Data scarcity
Pathology CDPM [31]	− Conditional architecture − Stable diffusion model − Pre-trained model	− High realism, pathological accuracy − Text-guided image synthesis	− Synthesizes central prostate part.
EMIT-DIFF [34]	− Controllable diffusion. − Text and edge information conditioning. − Pre-trained model.	− Text-guided image synthesis	− Low sampling speed
TDASD [36]	− Stable diffusion model: encoder (VAE), text encoder (CLIP), U-Net.	− Effective of limited data scenario	− Challenging with Fine-tuning on limited data.
GLIDE [32, 37]	− Stable diffusion model − Pre-trained model − Cross-attention layer	− Overcoming data scarcity.	− Requires extensive fine-tuning.

**Table 5:** Comparison of generative AI models employing transformers architecture for medical imaging: architectural designs, characteristics, and limitations.

**Model**\Refs.	**Architecture**	**Characteristics**	**Limitations**
TDCNN-Transformers [1]	− Transformer with autoencoder − CNN mapping feature	− Generating textual descriptions. − Generalization across image modalities.	− Quality of generated descriptions − Data availability
RRG-Transformer [41]	− Transformer with autoencoder. − CNN mapping feature.	− Used imaging with patient demographics. − Generate radiology reports.	− Reliance on available patient demographics
GAN-SIT [42]	− Conditional GAN − Spatial-Intensity Transform	− Visualizing changes in brain scans related to age and stroke severity.	− Requires fine-tuning.

**Table 6:** Overview of Text-to-Image generative AI models. The AE shows three tasks: (i) medical visual question answering (Med-VQA), (ii) medical image-text classification, and)iii) medical image-caption retrieval for both image-to-text and text-to-image.

**Method**		**Description**	**Dataset**		**Measure**		**Evaluation**		**Refs.**
GAN	DM-GAN	Develop the Story-Writer app for image generation. The DM-GAN tackles multilayering weaknesses and preserves image quality in the final layers.	MS-COCO		-				[12]
AEs	ELITE	Use the global map to encode an image with the prime and the secondary words for clarity. The local map focuses on extracting primary words.	Testset		CLIP-T (Text-alignment)		0.255		[31]
					CLIP-I (Image-alignment)		0.762		
					DINO-I		0.652		
	M3AE	Use the different masking ratios for images and text, leverage features from various layers for reconstruction, and employ distinct decoders for vision and language. Evaluated three tasks (Med-VQA, medical image-text classification, and medical image-text retrieval).		VQA-2019	Med-VQA	Acc	79.87		[21]
				MedlCaT	MedlCaT		Acc		
				ROCO	I2T	Recall_10_	65.80		
					T2I	Recall_10_	66.65		
		
Diffusion	Masked-Attention guidance	Enhances image generation fidelity to semantic masks, enabling precise text-guided editing and spatial accuracy.	COCO		FID		0.457		[15]
					mIoU		24		
	Multi-Modal MR	Use a basic framework, LDM, to capture the semantic features of input text descriptions with cross-attention layers. LDM integrates a U-Net architecture to reduce noise. Also, it integrates a large pre-trained language model, CLIP.	Brain MR (Privet) with 4- FLAIR, T1, T1Gd, and T2		FFID		FLAIR	43.23	[10]
							T1	46.58	
							T1Gd	45.47	
							T2	41.35	
	EMIT-DIFF	Use RadImageNet pre-training and fine-tuning datasets to improve medical image segmentation from text-guided.	Ultrasound Breast		Dice		71.65%		[36]
					Recall		81.91%		
			CT Spleen		Dice		94.78%		
					Recall		95.55%		
			MRI Prostate		Dice		83.98%		
					Recall		84.56%,		
	DPM	Utilize Stable-diffusion-based with text and image-based conditioning to generate realistic prostate MR.	Prostate MR (open source), Multi-Sequence Prostate MR (privet) Both with ADC and T2W Images		Acc		ADC	0.563	[34]
					Acc		T2W	0.625	

**Table 7:** Overview of Image-to-Text generative AI models

**Method**		**Description**	**Dataset**	**Measure**		**Evaluation**	**Refs.**
AEs	Attention-based AE	An attention-based encoder-decoder architecture. The encoder is a Resnet50 model, while the decoder is an LSTM model to refine the caption given from medical image by retrieving the most similar existing caption.	ROCO	BLEU-1		46.61	[32]
				BLEU-2		32.97	
				BLEU-3		23.63	
				BLEU-4		18.61	
Transformer	Based on CNN	A transformer-based encoder-decoder that encoder block encodes CNN features, while the decoder block takes as input a tensor containing text tokens and the outputs generated by the transformer encoder block.	ROCO	Accuracy		0. 762	[1]
				BLEU-1		0.538	
	Multi-Modal	CNN extracts the visual features from CXRs with semantic text embeddings with an encoder-decoder that connects visual features and embeds text to generate reports.	MIMIC-CXR, MIMIC-IV	BLEU-1		6.1%	[41]
				BLEU-2		8.0%	
				BLEU-3		1.4%	
				P_BERT_		11%	
				F1_Score_		17.4%	

**Table 8:** Overview of Image-to-Image generative AI models.

**Method**		**Description**	**Dataset**	**Measure**		**Evaluation**			**Refs.**
GANs	Cycle-GAN based-on attention gate	Generate high-quality synthetic skVCT images from MVCT data to improve treatment accuracy and facilitate adaptive radiotherapy based on attention gate and residual blocks.	41 slices of head and neck cancer	Compare kV-MV		MAE		109.6	[20]
						PSNR		27.5	
						SSIM		91.9	
				Compare kV-skV		MAE		60.6	
						PSNR		34.0	
						SSIM		96.5	
	Style-GAN	Use the same form structure without changing. Then apply U-Net to train the artificial Synthesis for segmentation.	ACDC	FID		24.74			[5]
				Dice		0.87			
			IDRiD	FID		1.09			
				Dice		0.80			
			SLiver07	FID		29.06			
				Dice		0.36			
	AttnGAN	Implement two generative models (single-GAN and multi-GAN) to compare the results. The G takes the input after the D extracts the features.	CUB 2011 bird	Acc	Single-Gan		78%		[24]
					Multi-Gan		84%		
Diffusion	Medfusion	The pre-trained autoencoder from the first phase encodes the image space into the latent space and the final decoded back into the image space. A U-Net model is trained to denoise. Images are finally generated with the DDIM.	AirOGS	FID		11.63			[22]
				Recall		0.40			
			CRCDX	FID		30.03			
				Recall		0.41			
			CheXpert	FID		17.28			
				Recall		0.32			

**Table 9:** A comprehensive summary of the datasets commonly utilized in medical imaging research for generative AI models, highlighting their primary characteristics, modalities, targeted organs, data splits, and specific tasks they are employed for.

**Dataset**\Refs.	**Description**	**Modality**	**Organ**	**Train/test**	**Task**
ACDC [5]	− 150 short-axis cardiac cine-MRI exams were conducted at the University Hospital of Dijon. − Dividing into five subgroups: four pathological and one healthy.	MRI	Heart	− 100 training: 1902 2D slices. − 50 testing: 1078 2D slices	− Data augmentation − Segmentation
SLiver07 [5]	− 40 CT volumes of the liver, enhanced with contrast agent. − Volumes are pathological with at least one tumor.	CT	Liver	− 20 training − 10 validations − 10 testing	− Data augmentation − Segmentation
IDRiD [5]	− 516 retinal fundus images of normal and pathological cases. − With disease grading ground truth and 81 segmentation masks.	Fundus	Retina	− 54 training (out of 81 with segmentation masks)	− Data augmentation − Segmentation
DDSM [13]	− 2500 breast molybdenum target images, with a focus on 210 selected cancer images. − Images display features like lumps or calcifications.	Mammography	Breast	− 170 cases for training − 40 cases for testing	− Data augmentation
E-ALL-IDB-I [14]	− Includes 108 images and 108 segmented images of RBC, WBC, and Platelets with total432. − Contains images of both healthy and non-healthy patients.	Microscopic	Multi-blood cells	− 20 validations.	− Data augmentation − Segmentation − Classification
E-ALL-IDB-II [14]	− Derived from ALL-IDB-I, contains 260 cropped images of WBCs. − Classification into Blast and Normal cells.	Microscopic	White blood cells	− 20 validations.	− Data augmentation − Segmentation − Classification
NPC-CT [20]	− Collected from 41 NPC patients who received HT. − KVCT and MVCT images used with registrations and transformations for analysis.	KVCT/MVCT	Head and Neck	− 25 training: 2995 slices. − 16 testing: 1898 slices	− Generate synthetic images
Kvasir-v2	Contains 8,000 endoscopic images across eight categories for disease classification in the digestive system.	Endoscopic	Digestive system	− 800 training (per class) − 200 validations (per class)	− Data augmentation − Classification
LIDC [27]	− Lung Image Database Consortium image collection	CT	Lung	− 90% training − 10% testing	− Generate longitudinal data
Breathing Motion [27]	− Consists of thoracic CT scans showing the breathing phases of lung cancer patients, − Used to model exhaling phases.	CT	Lung	− 90% training − 10% testing	− Generate longitudinal data
Tumor Regression [27]	− Contains daily treatment scans showing tumor regression during radiotherapy − Volumetric changes over time.	CT	Lung	− 90% training − 10% testing	− Generate longitudinal data
ROCO [1, 21, 32]	− Over 81,000 medical image-text pairs − Split to radiology class with over 65k images and out-of-class with 6,127 images. − covering a wide range of medical conditions and scenarios	Mixed	Various	− 60%training − 20% validations − 20% testing	− Classification − Image-to-texts − Text-to-image s − VQA
MedICaT [21]	− Over 217,000 medical images with captions and inline textual references. − A rich dataset for exploring image-text relationships.	Mixed	Various	− 1000 validations. − 1000 testing.	− Classification − Image-to-texts − Text-to-image s − VQA
DermaMNIST [30]	− 10,015 dermatoscopic images of various skin lesions.	Dermoscopic	Skin	Not specified	− Data augmentation − Classification
BloodMNIST [30]	− 17,092 blood cell images of healthy individuals. − Classify into eight distinct groups.	Dermoscopic	Blood cells	− Not specified	− Data augmentation − Classification
Glioma MRI [10]	− 484 glioma patients with annotations for isocitrate dehydrogenase (IDH) mutations. − Includes FLAIR, T1, T1Gd, and T2 modalities.	MRI	Brain	− Not specified	− Text-to-images
AIROGS [22]	− 101,442 RGB eye fundus images from about 60,357 subjects − Identifying referable and non-referable glaucoma cases.	Fundoscopic	Eye	− Not specified	− Data augmentation − Classification
CRCDX [22]	− 19,958 RGB histology images of colorectal cancer − Classified into microsatellite stable and microsatellite unstable.	Histology	Colorectal	− Not specified	− Data augmentation − Classification
CheXpert [22, 38]	− Over 223,414 Gray-scaled chest radiographs of over 64,534 patients. − Associated radiology reports. − Some classifications for cardiomegaly.	X-ray	Chest	− 70% training − 30% testing	− Data augmentation − Classification − Text-to-images
Open-source Prostate MR [34]	− 1,095 3D prostate MR images. − With 32 2D-slices for both ADC and T2W.	MRI	Prostate	− 10 central slices training (from each 3D image) − 200 cases evaluations	− Text-to-images
Close-source Prostate MR [34]	− Multi-parametric 3D MR images from 850 patients. − Lesions manually contoured by a radiologist. − Includes T2W and DW images.	MRI	Prostate	− 10 central slices training (from each 3D image) − 200 cases evaluations.	− Text-to-images
HAM10000 [34]	− 10,015 dermoscopic images annotated for seven skin lesions.	Dermoscopic	Skin	− 80% training − 20% testing	− Text-to-images
RadImageNet [36]	− Large-scale medical imaging with different modalities. − 1.35 million radiological images.	− MRI − CT − Ultrasound	Various	− 3-fold cross-validation	− Text-to-images − Segmentation
MIMIC-CXR [38, 41]	− Over 227,835 imaging chest radiographs of over 65,379 patients. − Associated radiology reports.	− X-ray	Chest	− 70% training − 30% testing	− Text-to-images
MIMIC-IV [41]	− Demographic information encompasses gender, age, and ethnicity related to patients. − Used alongside medical images for enhancing radiology report generation.	− Non-imaging	Various	− 70% training − 30% testing	− Image-to-texts
ADNI [42]	− Longitudinal T1-weighted MRIs of subjects with varying stages of neurodegeneration, − Conditioned on age, baseline diagnosis, and cognitive scores.	− MRI	Brain	− 3228 training − 749 testing	− Generate synthetic images
MRI-GENIE [42]	− Clinical quality MRIs from stroke patients. − Conditioned on age and disease severity.	MRI	Brain	− 334 training − 1403 testing.	− Generate synthetic images

## LIST OF ABBREVIATIONS

DL: Deep learning
GANs: Generative adversarial networks
CT: Computed tomography
MRI: Magnetic resonance imaging
AEs: Autoencoders
LDM: Latent Diffusion Model
MSE: Mean square error
WBCs: White blood cells
RBCs: Red blood cells
OT: Optimal transport
DDIM: Denoising diffusion implicit model
DPM: Diffusion probabilistic model
SIT: Spatial-Intensity Transform
SITs: Spatial-Intensity Transforms
IoT: Internet of Things
